# Prodromal Parkinsonian Features in Carriers of Gaucher Disease Compared to Controls

**DOI:** 10.3390/life15060952

**Published:** 2025-06-13

**Authors:** Michal Becker-Cohen, Ari Zimran, Tama Dinur, Maayan Tiomkin, Arndt Rolfs, David Arkadir, Peter Bauer, Elena Shulman, Gilad Yahalom, Mikhal E. Cohen, Orly Manor, Ora Paltiel, Shoshana Revel-Vilk

**Affiliations:** 1 Gaucher Unit, The Eisenberg R&D Authority, Shaare Zedek Medical Center, Jerusalem 9103102, Israel; galitb@szmc.org.ildinurt@szmc.org.il (T.D.); matiomkin@gmail.com (M.T.); elename@szmc.org.il (E.S.); svilk@szmc.org.il (S.R.-V.); 2 Braun School of Public Health and Community Medicine, Hebrew University of Jerusalem, Jerusalem 9124001, Israel; orlyma@ekmd.huji.ac.il (O.M.); orap@hadassah.org.il (O.P.); 3 Agyany Pharma Ltd., Jerusalem 9695614, Israel; arndt.rolfs@agyanypharma.com; 4 Faculty of Medicine, Hebrew University of Jerusalem, Jerusalem 9124001, Israel; arkadir@hadassah.org.il (D.A.); gilady@szmc.org.il (G.Y.); 5 Centogene GmbH, 18055 Rostock, Germany; peter.bauer@centogene.com; 6 Medical Faculty, University of Rostock, 18051 Rostock, Germany; 7 Rare Disease Consulting RCV GmbH, Leibnizstrasse 58, 10629 Berlin, Germany; 8 Department of Neurology, Hadassah Medical Center, Hebrew University of Jerusalem, Jerusalem 9124001, Israel; 9 Department of Neurology, Shaare Zedek Medical Center, Jerusalem 9103102, Israel; mikhalc@szmc.org.il

**Keywords:** Parkinson’s disease, Gaucher disease, GBA1 carriers, prodromal Parkinson’s disease, Sidransky syndrome, principal component analysis

## Abstract

Carriers of Gaucher disease have an increased risk of developing Parkinson’s disease (PD). Identifying PD in its prodromal stage is crucial, as early detection before motor symptoms appear allows for potential interventions to salvage neurons and slow or prevent disease progression. At the Gaucher unit at Shaare Zedek Medical Center, we are following a large cohort of obligatory carriers of GBA1 variants (GBA1 carriers) and study ways to identify those at an increased risk for developing PD. In this study, we compared non-invasive prodromal PD tests in 164 GBA1 carriers and 49 participants with no genetic predisposition to PD (controls). The proportion of abnormal tests was compared between groups, and the risk factors for having abnormal tests (at least one or ≥20%) were studied. There were no differences between GBA1 carriers and controls in the frequency of abnormalities, having at least one abnormal test or having ≥20% abnormal tests. Having ≥20% of abnormal tests was associated mainly with age. Principal component analysis identified distinct cognitive, motor, and non-motor dysfunction patterns in GBA1 carriers compared to controls, with cognition in GBA1 carriers more closely linked to motor dysfunction and less influenced by mood and sleep, while in controls, executive function was tied to emotional state and fatigue. Younger carriers outperformed older ones in motor and some cognitive tasks. Those with a family history of PD showed worse cognitive scores than participants with no family history. Sex-based analysis revealed males obtained higher scores in most of the cognition subtests of the NeuroTrax test, whereas it was females in motor and other cognitive domains, mainly in the group of GBA1 carriers. A longitudinal follow-up of GBA1 carriers is ongoing to understand PD progression in GBA1 carriers with the aim of offering targeted intervention for those at higher risk.

## 1. Introduction

Gaucher disease (GD) is a genetic disorder caused by variants in the *GBA1* gene, leading to a mutant glucocerebrosidase enzyme. This enzyme fails to fully hydrolyze its substrate glucocerebroside and, therefore, leads to its accumulation in the lysosomes, mainly in macrophages. In addition, the genetic change(s) lead to misfolding of the mutant enzyme, which causes a series of pathological consequences in neurons, also observed among carriers [1,2]. Clinically, GD presents with a range of symptoms, including cytopenias, hepatosplenomegaly, bone involvement and, in severe forms, neurological impairments.

Parkinson’s disease (PD) is a progressive neurodegenerative disorder marked by the depletion of dopamine-producing neurons in the substantia nigra, presenting with symptoms such as tremors, rigidity, bradykinesia, and postural instability.

Individuals with monoallelic or biallelic *GBA1* variant (patients with GD) show an increased risk for developing PD [3]. Conversely, approximately 10–15% of patients with PD carry at least one *GBA1* variant, a condition referred to as GBA1-related PD [4] or, as recently suggested, ‘Sidransky syndrome’ [5]. This form of PD is more severe than the more common, non-genetic (idiopathic) PD and is characterized by an early age of onset, poor response to treatment, and significant cognitive decline, although there are wide variations in clinical presentation [3]. A meta-analysis published in 2009 showed that those carrying the mild N370S variant (recently referred to as c.1226A > G; p.N409S, yet, since the older nomenclature is more widely used, the new one will not be implemented herein) had a three-fold risk of developing PD, while carriers of other *GBA1* variants had a significantly higher (10–15-fold) risk compared to the non-carrier population [6,7].

Prior to the typical motor symptoms of PD, there is a pre-motor phase known as prodromal PD, which can be as long as 10 to 20 years. This phase has mostly autonomic presentations, such as anosmia, sleep disorder, constipation, postural hypotension and depression [8,9]. PD is clinically diagnosed once the typical motor symptoms appear. There is a consensus that at least 60% of dopaminergic neuron loss is needed for the motor symptoms to become apparent, and it may be too late to salvage any functioning neurons. Therefore, there is a strong rationale for identifying PD in its prodromal stage, before the motor symptoms appear, in attempts to slow or even prevent the inevitable PD progression. At present, there are no treatments that can alter the disease course, and all therapeutic modalities are limited to symptomatic care. Recently, several clinical trials have been initiated, also targeting the prodromal PD phase, when many cells are not yet dead, and, hence, the reversibility of dopaminergic neuron dysfunction may occur [3]. The realization that many of the dopaminergic neurons are not dead but injured and dysfunctional is inspired by the reversibility of some of the neuronopathic features in type 3 and type 2 GD (caused by the very same misfolded enzyme [10,11]), as documented by the animal model of transgenic drosophila fruit flies [12], and this is the basis for the ongoing clinical trials using high-dose ambroxol in GBA-related PD [3].

Although the existence of prodromal PD is acknowledged, it is not yet commonly ascertained/evaluated clinically. The International Parkinson and Movement Disorder Society (MDS) has developed a set of research criteria for prodromal PD, published in 2015 and updated in 2019 [8,13]. These criteria include risk factors, such as sex, age, family history of PD, known PD-related genetic variants, environmental factors (such as exposure to pesticides and solvents, caffeine consumption, and smoking habits), urinary and erectile dysfunction, and assessments of motor and non-motor clinical symptoms.

In 2017, we launched a screening study for prodromal PD features in 98 participants who are carriers of *GBA1* variants (GBA1 carriers) in order to identify those with prodromal PD findings. Using non-invasive tests to assess different domains of PD, we found >15% of “abnormal” tests in around 20% of GBA1 carriers [9]. In the current study, we use the same set of non-invasive tests to compare GBA1 carriers to participants with no genetic predisposition to PD in order to highlight potential differences and frequencies of prodromal features between both groups and possibly identify those individuals with a higher rate of PD-related prodromal features that would render these individuals eligible for future prevention studies.

## 2. Methods

### 2.1. Study Population

Patients with GD, family members of patients with GD and healthy volunteers, none of whom had PD, between the ages of 35 and 80 were enrolled in this study. Since recruitment for a comprehensive study for prodromal PD is challenging, and even more so for individuals without a genetic risk, the selection of all subjects for this study was based on convenience rather than pre-identified power analysis.

Blood samples were applied onto a filter card and sent to Centogene, Rostock, Germany, for PD panel (sequencing of 68 PD-related genes, including *GBA1*) [4]. Previously, we published a study testing prodromal PD in a cohort of 98 carriers of a single *GBA1* variant (GD carriers). For the current study, we added 25 GD carriers and 41 patients with GD and defined the participants as the GBA1-carrier group. The N370S *GBA1* variant was marked as mild and all other *GBA1* variants as severe. For patients with GD [with two *GBA1* variants], those who were homozygous for the N370S variant were marked as mild, and all other *GBA1* variant combinations were marked as severe. Those with normal *GBA1* sequencing were defined as controls. Sequencing of the other 67 PD-related genes revealed two participants from the GBA1-carrier group and one from the control group carrying a pathogenic variant in the LRRK gene (c. 6055G > A p.Gly2019Ser). Those participants were excluded from the study.

Participants enrolled after December 2019 were questioned at enrollment visit about risk factors associated with PD, including coffee and tea consumption, smoking habits, exposure to solvents and pesticides, and family history of PD (1st- and 2nd-degree relatives). Participants enrolled prior to this date (enrollment initiated in May 2017) were contacted by telephone to complete this questionnaire.

The institutional review board (IRB) of Shaare Zedek Medical Center (SZMC) approved the study design (NCT05253560), and all participants provided two written informed consent forms, one for performing all study procedures and one specific for the genetic testing.

### 2.2. Prodromal PD Features Evaluation

All participants underwent pre-defined, non-invasive tests for assessing prodromal PD, as previously described [9]. Three study coordinators (MBC, TD, and ES) administered the prodromal PD testing, and each oversaw the same group of tests.

**Figure 1:**
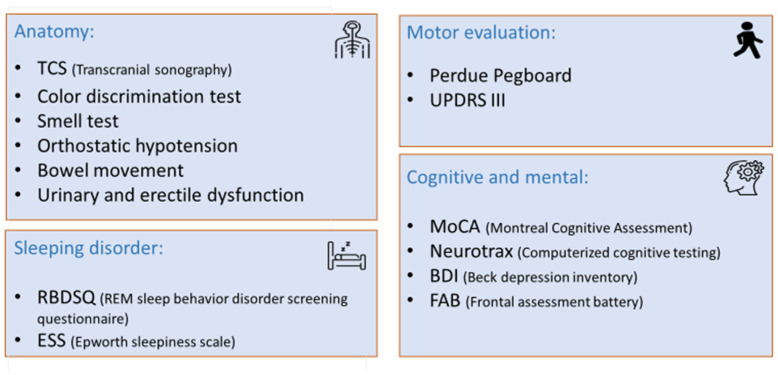
Non-invasive prodromal test divided into four domains.

The prodromal tests, which aimed to assess different aspects of PD, were divided into four domains: imaging, sensory and autonomic, cognitive and mental, sleeping disorder, and motor evaluation (Figure 1). Each test is described together with the literature-based cutoff [9].

The imaging domain was evaluated by transcranial sonography (TCS), testing the hyper-echogenicity of the substantia nigra in the midbrain by a single imaging technician (MT) using the Philips/HP Sonos 5500 ultrasound, S4/2.0–2.5 (Davis Medical Electronics, Inc., Vista, CA, USA). A cutoff value ≥ 0.2 cm^2^, found in more than 90% of patients with PD, was considered abnormal (sensitivity 90.7%, specificity 82.4%, and positive predictive value 92.9%).

To evaluate the sensory and autonomic domain, we used five separate tests: smell (hyposmia), orthostatic hypotension, color discrimination, constipation, and urinary and erectile dysfunction. Hyposmia was evaluated using the B-SIT^®^ version (12 smell card) (Sensonics International, Haddon Heights, NJ, USA) of the University of Pennsylvania Smell Identification Test. A score of <8 was considered abnormal. Orthostatic hypotension (OH) was defined as a difference of at least 20 mmHg systolic or 10 mmHg diastolic blood pressure after 3 min between sitting and standing. Color discrimination was analyzed using Farnsworth-Munsell 100 Hue Color Vision Test (Luneau Technology (a.k.a. Visionix) Headquarters, Pont-de-l’Arche, Normandie, France). The 95% CI normal for age of the total error score (TES) was used for analysis. Participants were asked about their weekly bowel movement frequency; a frequency of under 0.5 per day was rated as abnormal (constipation). Urinary and erectile dysfunction was assessed according to the MDS criteria.

The cognitive and mental section was evaluated using four separate tests: the Montreal Cognitive Assessment (MoCA) (mocacognition.com (accessed on 10 June 2025)), NeuroTrax^TM^ (NeuroTrax Corp., Medina, NY, USA), Beck Depression Inventory (BDI) (https://www.ismanet.org/doctoryourspirit/pdfs/Beck-Depression-Inventory-BDI.pdf (accessed on 10 June 2025)) and Frontal Assessment Battery Test (FAB) (https://psychscenehub.com/wp-content/uploads/2018/07/Frontal_FAB_Scale.pdf (accessed on 10 June 2025)). MoCA was evaluated using a tool for early detection of mild cognitive impairment. A score of ≤25 was considered abnormal. NeuroTrax^TM^ is a computerized battery test that assesses brain wellness and includes seven sections: memory, executive function, attention, information processing speed, visual–spatial, verbal function, and motor skills. A score ≤85th percentile (-SD) was considered abnormal, adjusted for age, sex and level of education. BDI, a self-administered questionnaire, includes 21 questions used to screen, diagnose, and measure the severity of depression. A cutoff of ≥14 indicated depression. FAB is a screening test for frontotemporal dementia. A score of ≤16 was considered abnormal.

To assess sleep behavior, we used two self-administered questionnaires: the rapid eye movement (REM) sleep behavior screening questionnaire (RBDSQ) (https://eprovide.mapi-trust.org/instruments/rem-sleep-behavior-disorder-screening-questionnaire (accessed on 10 June 2025)), where a score of ≥5 was considered abnormal, and daytime sleepiness using the Epworth sleepiness scale (ESS) (https://www.cdc.gov/niosh/work-hour-training-for-nurses/longhours/mod2/epworth-P.pdf (accessed on 10 June 2025)), where a score of >10 was considered pathological sleepiness.

We assessed the motor section using two tests: the Unified Parkinson’s Disease Rating Scale (UPDRS)-part III measuring gross motor function, where a score >6, excluding postural and action tremors, was considered abnormal, and Perdue pegboard test, which measures the fine motor skills, average placement of <11 pegs at 30 s using both hands.

### 2.3. Statistical Analysis

The Shapiro–Wilk normality test, used to test for normality, showed a skewed distribution of the variables; thus, to report the descriptive statistics summary, we used the median (range) for continuous variables. For nominal data, we reported absolute and relative frequencies. Risk factors for PD in GBA1 carriers and controls were compared using the Mann–Whitney test for continuous variables and the chi-square test for categorical variables.

#### 2.3.1. Rate of Abnormal Prodromal Tests

To study the frequency of each test abnormality, we categorized the test result as normal or abnormal. Six different cutoff scores, as described below, were used in separate analyses. The abnormality rate of each test was calculated based on the number of participants with abnormal values divided by the number of participants who performed the test. A chi-square test was used to compare the abnormality rate for each test between GBA1 carriers and controls.

The cutoffs used for this study were as follows:

- The cutoff defined in the literature [9].
- 10th percentile—the top, worst 10% of each test in the controls ordered from best to worst.
- 25th percentile—the top, worst 25% of each test in the controls ordered from best to worst.
- Outliers—scores of >1.5 IQR (Inter Quartile Range) from Q3 (the third quartile) in the box plot values for the controls.
- Above 1.64 z-score—each test in the GBA1 carriers group was converted to z-score values, and the cutoff value was determined above 1.64 SD.
- Above 1.96 z-score—each test in the GBA1 carriers group was converted to z-score values, and the cutoff value was determined above 1.96 SD.

Due to multiple testing rounds, a significance level of α < 0.01 was considered statistically significant.

#### 2.3.2. Risk Factors for Having Abnormal Prodromal Tests

To evaluate the risk factors associated with having abnormal prodromal tests, we used the first definition, i.e., literature based. We then calculated the percentage of “abnormal” tests for each subject from the total tests they performed. We compared those with no abnormal tests to participants with mild abnormality tests (at least one abnormal test and up to 20%) and to participants with severe abnormality, defined as over 20% abnormal tests. The following variables were entered into the model: *GBA1* status, age, sex, family history of PD, smoking history, coffee intake, and exposure to solvents and pesticides.

#### 2.3.3. Principal Component Analysis

As our prodromal evaluation included a wide range of tests, we examined the possibility of aggregating the tests into a smaller number of factors using principal component analysis (PCA), which has the potential to increase interpretability and enables us to reduce dimensionality of the dataset, while, at the same time, minimizing information loss. We limited the number of components to three and analyzed each group separately, i.e., GBA1 carriers and controls. We then compared the components identified for GBA1 carriers vs. the controls.

We used the three components identified for each group, i.e., GBA1 carriers and controls, to compare different subgroups: male vs. female, with family history of PD compared vs. without, and age over 55 vs. younger. In GBA1 carriers, additional subgroups were compared: mild vs. severe variants and patients with GD vs. carriers of one *GBA1* variant. These comparisons were carried out using *t*-tests. These tests are less affected by multiple comparisons, as the number of compared variables is substantially smaller (3 components compared with 16 tests).

As the PCA cannot analyze all the data in the case of missing values, we checked for missing data and used multiple imputation method to replace the missing values. The median (range) of tests performed by each participant was 97% (78–100%). Reasons for missing data included participants unable to complete the full four-hour testing session despite prior notification, incomplete UPDRS motor assessment due to personal preference, incomplete smell assessment due to total anosmia and incomplete color discrimination test due to color blindness. We generated five imputed datasets using a probabilistic model, and each dataset was analyzed separately. After reviewing the five models, which showed very similar results, we randomly selected the fifth for analysis. This multiple imputation method enhances the accuracy of estimates and standard errors, making it more reliable.

Statistical analysis was performed using SPSS version 26.0.

## 3. Results

### 3.1. Participant Characteristics

A total of 213 participants (99 male; 46.5%) were recruited to this study at a median (range) age of 53 (36–78) years, of whom 164 (77%) were GBA1 carriers and 49 non-GBA1 carriers (controls) (Table 1). All GBA1 carriers were either patients with GD or family members of patients with GD. As for the control group, 9 participants were family members of patients with GD; 36 were either spouses or friends of patients with GD; and 4 others had no relation to patients with GD. All controls tested negative for the *GBA1* variant and for any of the 67 additional PD-related genes. The median age of GBA1 carriers was lower compared to controls, but this did not reach clinical significance (Table 1). Controls were more likely to report exposure to solvents (Table 1). All other risk factors associated with PD did not differ between the two groups

Most carriers of GD were found to have the mild N370S variant (Table 2). Similarly, most patients with GD were homozygous to this variant.

### 3.2. Rate of Abnormal Testing in GBA1 Carriers Compared to Controls

No differences were found in the frequency of abnormal prodromal tests between the two groups, using the six abnormal test definitions described in the Methods. A comparison based on the literature cutoff and continuous measurement (when applicable) is shown in Table 3. A comparison based on the other five abnormal test definitions is shown in Supplementary Table S1a–d.

### 3.3. Risk Factors for Abnormal Prodromal Tests

At least one abnormal test was detected in 130 (79.3%) GBA1 carriers and 38 (77.6%) controls. Participants with at least one abnormality were significantly older, with a median age of 53 (range 36–78), compared to a median age of 46 (range 39–70) in those without abnormalities (*p* = 0.002).

A high frequency of abnormalities (≥20% abnormal tests) was found in 27 participants (12.7%), with no difference between GBA1 carriers and controls (Table 4). Older age and exposure to pesticides and solvents were found to be associated with the rate of abnormal tests.

The frequency of abnormal prodromal testing in GBA1 carriers was not associated with the type of *GBA1* variant (mild vs. severe), whether they were mono- or bi-allelic carriers, or with age. In controls, it was not linked to family history of PD.

### 3.4. Patterns of Principal Component Analysis and Percentage of Variance Explained by Components

The structure and loading patterns of the PCA differed between GBA1 carriers and controls (Table 5 and Table 6). In GBA1 carriers, the first component mostly represents executive and cognitive processing; the second component represents a combination of memory, cognition and motor function; and the third component represents mood, sleep, and transcranial ultrasonography (Table 5). In controls, the first component represents mood, sleep and cognitive processing; the second component mainly represents cognition and a deficit in color discrimination with high loading for MoCA, memory, and visual–spatial processing; and the third component mainly represents motor and autonomic functions (Table 6).

A comparison of the PCA between subgroups in GBA1 carriers and in controls is shown in Supplementary Table S2. The three components identified in GBA1 carriers were not associated with the severity of the *GBA1* variant nor with being a patient with GD vs. a carrier of GD. Younger GBA1 carriers had better motor and some cognitive performance (PCA2) compared to older GBA1 carriers. Those without family history of PD performed better in cognitive scores (PCA1) than participants with no family history. Sex was associated with all three components tested. The performance of males in the cognitive component was higher than female performance, and females performed better in other cognitive tests, motor function, and presented higher depression and mood scores. The three components in controls were not associated with sex or family history of PD. Younger controls had better performance in cognition (PCA2) and motor/autonomic (PCA1) compared to older participants.

## 4. Discussion

This study compared the proportion of abnormal prodromal PD features between GBA1 carriers and controls who underwent extensive testing. Unexpectedly, no significant differences were found between the groups across multiple dimensions, regardless of the threshold used to define abnormality.

Several factors may account for this finding. First, although GBA1 variants increase the risk of PD, most carriers will never develop the disease. Consequently, their prodromal test findings may not differ significantly from those of non-carriers. Additionally, most GBA1 carriers in our study had the mild N370S variant, which has a lower estimated conversion rate to PD (approximately 3–5-fold higher) compared to the non-N370S carriers (5–15-fold higher) [6,7], making detection of a statistically significant difference from the control population (risk of ~1% over age of 60 years) more challenging.

Second, a positive family history of PD in controls was similar to GBA1 carriers. Since family history is an independent risk factor for PD [8,13], this shared characteristic introduces selection bias that may have contributed to the lack of significant differences in the frequency of abnormal prodromal features.

Third, age-related changes in movement, cognition, and autonomic function can resemble early PD symptoms [14], making it difficult to distinguish between true prodromal features and aging. Thus, the older age of the control group in our study may have an increased rate of abnormal prodromal features similar to the level of prodromal features in GBA1 carriers.

Fourth, environmental factors, particularly solvent exposure, are a known risk factor for PD [15]. According to the MDS criteria, solvent exposure increases the likelihood of prodromal PD by a factor of 1.5 [8]. In our study, controls reported a higher rate of solvent exposure, which also explains the higher-than-expected prevalence of prodromal features. This was particularly noted as the only statistically significant risk factor associated with having at least 20% abnormal test results. These findings suggest that environmental influences on prodromal features may be more substantial than previously assumed and similar to genetic predisposition.

To better understand underlying patterns across prodromal tests, we conducted a PCA to reduce the number of variables and identify meaningful clusters of abnormalities. Our findings revealed distinct differences in how cognitive, motor, and non-motor functions were structured in GBA1 carriers compared to controls. In GBA1 carriers, cognitive function was independent of mood and sleep dysfunction, whereas in controls, executive function was closely linked to mood and sleep. This suggests that, under normal conditions, cognition is more dependent on emotional state and fatigue levels. In contrast, in GBA1 carriers, cognitive function was more closely associated with motor dysfunction, potentially indicating early neurodegenerative changes [16].

While most GBA1 carriers will not develop PD, the distinct clustering of cognitive and motor components in this group resembles early PD pathology. In GBA-related PD, cognitive decline usually proceeds motor symptoms but later becomes intertwined with cognitive decline [17]. Similarly, our findings suggest that in GBA1 carriers, subtle motor changes may already be linked to cognitive function, whereas in controls, motor skills remain separate from cognition.

Although this study represents the largest cohort of GBA1 carriers examined to date, the sample sizes of the two groups were still limited to detect subtle differences in test performance. Nevertheless, due to the consistency of the findings across the different domains, we assume that even an increase in the sample size will not lead to different conclusions. It is to be hoped that our ongoing longitudinal follow-up study will shed more light on the potential impact of specific prodromal features on the actual risk of developing GBA1-related PD and, accordingly, to the selection of candidates for interventional therapies.

## 5. Conclusions

This study found no significant differences in the rate of abnormal prodromal PD features between GBA1 carriers and controls, with age emerging as the primary predictor of abnormalities. Environmental factors, particularly solvent exposure, also played a role. Principal component analysis revealed distinct cognitive–motor relationships in GBA1 carriers, suggesting early neurodegenerative changes. Subgroup analyses highlighted cognitive differences in the GBA1-carrier group based on family history and sex-related variations. A longitudinal follow-up of GBA1 carriers is ongoing to understand PD progression in GBA1 carriers with the aim of offering targeted intervention for those at higher risk.

## Figures and Tables

**Table 1:** Risk factors associated with Parkinson’s disease in GBA1 carriers and controls.

	GBA-1 Carriers	Controls	*p*-Value
**Number**	164	49	
Male	76 (46%)	23 (47%)	0.94
Age, median (range)	51.5 (36–75)	56 (38–78)	0.07
Relatives with PD	47 (28.8%)	14 (28.6%)	0.99
1st relative	32	8	
2nd relative	21	8	
Caffeine use *	125 (86.2%)	31 (91%)	0.436
Smoking *			0.401
Current	14 (9.6%)	3 (8.8%)	
Former	31 (21.2%)	5 (14.7%)	
Never	101 (69.2%)	26 (76.5%)	
Regular pesticide exposure *	7 (4.8%)	2 (5.9%)	0.681
Solvent exposure *	8 (5.5%)	8 (23.5%)	0.003

PD, Parkinson disease; * answered by 146 GBA1 carriers and 34 controls since the risk factor questionnaire was introduced after the study began.

**Table 2:** The representation of the GBA1 variants in carriers and patients with GD (n = 164).

Carriers of GD	n = 123	Patients with GD	n = 41
N370S/wt	87 (71%)	N370S/N370S	28 (68%)
84GG/wt	13 (11%)	N370S/84GG	4 (10%)
L444P/wt	9 (7%)	N370S/L444P	2 (5%)
R496H/wt	4 (3%)	N370S/R496H	0 (0%)
V394L/wt	5 (4%)	N370S/V394L	2 (5%)
Other/wt	5 (4%)	N370S/Other	5 (12%)

GD, Gaucher disease; wt, wild type.

**Table 3:** Prodromal features in GBA1 carriers compared to controls.

Prodromal Feature	Literature Cutoff **	GBA1 Carriers	Controls	*p*-Value
Transcranial ultrasonography, cm^2^	≥0.2	30 (19.5%)	12 (26.1%)	0.41 *
	Median (range)	0.125 (0–0.42)	0.135 (0–0.46)	0.45 *
Color discrimination test (TES)	40–49 years >100	2 (1.3%)	0	0.99
	50–59 years >130			
	60–69 years >170			
	70–79 years >195			
	Median (range)	40 (0–171)	45.5 (8–154)	0.59
UPSIT—smell test	<8	23 (14.4%)	6 (12.5%)	0.94
	Median (range)	10 (0–12)	10 (6–12)	0.72
Orthostatic hypotension	>20 SBP or >10 mmHg DBP	30 (21%)	10 (25%)	0.66 *
Bowel movement (daily)	≤0.5	10 (6.4%)	5 (10.6%)	0.36
Urinary dysfunction	Yes/No	23 (16%)	5 (14.7%)	0.99 *
Erectile dysfunction	Yes/No	5 (9.4%)	0 (0%)	0.57 *
Beck depression inventory	≥14	15 (9.9%)	3 (6.4%)	0.57 *
	Median (range)	4 (0–25)	4 (0–16.5)	0.78 *
Frontal assessment battery	<16	1 (0.7%)	0	0.99 *
	Median (range)	18 (15–18)	18 (16–18)	0.26 *
MoCA—Total score	≤25	37 (23.9%)	9 (20%)	0.67 *
	Median (range)	27 (21–30)	27 (22–30)	0.63 *
MoCA—Visuospatial/executive	≤3	38 (24.8%)	10 (21.7%)	0.75
	Median (range)	4 (2–5)	4 (2–5)	0.65 *
Neurotrax—Memory	<85	12 (7.2%)	4 (8.2%)	0.93
	Median (range)	104.5 (61.1–115.7)	102.5 (68.8–111.8)	0.57
Neurotrax—Executive function	<85	6 (3.6%)	2 (4.1%)	0.99
	Median (range)	107.5 (77.7–134.2)	107.9 (72.3–119)	0.48
Neurotrax—Attention	<85	5 (3%)	2 (4.1%)	0.93
	Median (range)	104.6 (63.5–119.4)	104.2 (70.7–116.5)	0.36
Neurotrax—Information processing speed	<85	15 (9.1%)	4 (8.2%)	0.51
	Median (range)	102.95 (25–150.1)	104.6 (25–133.7)	0.75
Neurotrax—Visual spatial	<85	19 (11.4%)	5 (10.2%)	0.81
	Median (range)	105.4 (59–138.6)	108.9 (77.70–133.40)	0.64
Neurotrax—Verbal function	<85	13 (7.9%)	8 (16.3%)	0.14
	Median (range)	104.2 (25–116.4)	101.8 (25–114.6)	0.01
Neurotrax—Motor skills	<85	5 (3%)	4 (8.2%)	0.25
	Median (range)	108.4 (25–120.8)	106.7 (25–121)	0.14
Neurotrax—Global cognitive score	<85	4 (2.4%)	1 (2%)	0.99
	Median (range)	105.15 (75.20–121.3)	103.80 (79.80–116.4)	0.38
REM sleep behavior disorder	≥5	18 (11.7%)	11 (22.9%)	0.15
	Median (range)	2 (0–12)	2 (0–7)	0.49 *
Epworth sleepiness scale	>10	24 (15.1%)	4 (8.3%)	0.34
	Median (range)	6 (0–20)	6 (1–20)	0.48 *
Purdue pegboard	<11	2 (1.3%)	0 (0%)	0.99
	Median (range)	21 (9.7–32.3)	20.8 (12.7–30.3)	0.79 *
UPDRS-III	>10	7 (5%)	1 (2.5%)	0.81
	Median (range)	2 (0–18)	2 (0–12)	0.01 *

TES, total error score; UPSIT, University of Pennsylvania Smell Identification Test; MoCA, Montreal cognitive assessment; REM, Rapid eye movement; UPDRS, Unified Parkinson’s Disease Rating Scale. * adjusted for age (for tests not age-adjusted); ** cutoff scores as previously described [9].

**Table 4:** Risk factors associated with the rate of abnormal prodromal tests.

	No Ab Tests	<20% Ab Tests	≥20% Ab Tests	*p*-Value
Number	45	141	27	
Age, median (range)	46 (39–70)	53 (36–78)	58 (40–73)	0.004
Carriers	34 (75.6%)	111 (78.7%)	19 (70.4%)	0.74 **
Male	15 (33.3%)	71 (50.4%)	13 (48.1%)	0.12 **
Relatives with PD	13 (29.5%)	39 (27.7%)	9 (33.3%)	0.813 **
Caffeine use *	8 (18.2%)	24 (17%)	8 (29.6%)	0.23 **
Smoking *				0.93 **
Current	4 (11.1%)	10 (8.1%)	3 (14.3%)	
Former	7 (19.4%)	26 (21.1%)	3 (14.3%)	
Never	25 (69.4%)	87 (70.7%)	15 (71.4%)	
Regular pesticides exposure *	0	6 (4.9%)	3 (14.3%)	0.02 **
Solvent exposure *	3 (8.3%)	7 (5.7%)	6 (28.6%)	0.04 **

Ab, abnormal; PD, Parkinson disease; * answered by 146 GBA1 carriers and 34 controls since the risk factor questionnaire was introduced after the study began. ** adjusted for age.

**Table 5:** Principal component analysis: component loading pattern for GBA1 carrier.

GBA1 Carriers			
Component	Component 1	Component 2	Component 3
Domain	Cognitive	Cognitive and Motor	Sleep and Cognitive
Tests	Executive function (0.804)	MoCA (0.590)	TCS (0.431)
	Attention (0.768)	FAB Score (0.553)	Verbal function (0.366)
	Information processing speed (0.785)	Memory (0.456)	ESS (−0.514)
	Visual spatial (0.483)	Both hands (0.625)	BDI (−0.706)
	Motor skills (0.535)	Colour discrimination (−0.747)	RBD (−0.633)
		Smell test (0.351)	
		UPDRS (−0.384)	
Variance explained	23.40%	11.10%	8.70%

The parentheses indicate the weight of contribution of each variable to the principal component (minus loading is a negative impact of the variable on the component). MoCA, Montreal cognitive assessment; FAB, frontal assessment battery; UPDRS, Unified Parkinson’s Disease Rating Scale; TCS, Transcranial ultrasonography; ESS, Epworth sleepiness scale; BDI, Beck depression inventory; RBD, Rapid eye movement sleep behavior disorder.

**Table 6:** Principal component analysis: component loading patterns for controls.

Control			
Component	Component 1	Component 2	Component 3
Domain	Cognitive, Sleep & Mood	Cognition	Motor & Autonomic Functions
Tests	Executive function (0.660)	MoCA (0.772)	Both hands (0.603)
	Attention (0.690)	Memory (0.828)	Bowel movements (0.598)
	Information processing speed (0.529)	Visual spatial (0.705)	Motor skills (0.316)
	FAB Score (0.601)	Verbal function (0.455)	UPDRS (−0.556)
	ESS (−0.563)	Colour discrimination (−0.448)	TCS (−0.754)
	BDI (−0.542)		Smell test (0.304)
	RBD (−0.554)		
Variance explained	20.60%	12.60%	10.10%

The parentheses indicate the weight of contribution of each variable to the principal component (minus loading is a negative impact of the variable on the component). FAB, frontal assessment battery; ESS, Ep-worth sleepiness scale; BDI, Beck depression inventory; RBD, Rapid eye movement sleep behavior disorder; MoCA, Montreal cognitive assessment; UPDRS, Unified Parkinson’s Disease Rating Scale; TCS, Transcranial ultrasonography.

## Data Availability

All data are kept at the Gaucher unit according to GCP and institution regulations.

## Supplementary Materials

The following supporting information can be downloaded at: https://www.mdpi.com/article/10.3390/life15060952/s1, Table S1: Rate of abnormal prodromal tests in GBA1 carriers compared to controls according to six different cutoff scores; Table S2: PCA comparing subgroups in GBA1 carriers vs. controls.
