# Supplementary material for: Prodromal Parkinsonian Features in Carriers of Gaucher Disease Compared to Controls
Source: Life (Basel). 2025 Jun 13;15(6):952. doi: 10.3390/life15060952 (PMC12194337; doi:10.3390/life15060952)
Supplement: Supplementary file 1 [file life-15-00952-s001.zip › Supplement Table S1.pdf]

Table S1: Rate of abnormal prodromal tests in GBA1 carriers compared to controls according to six different cutoff scores

(a)Transcranial sonography, sensory and autonomic assessments		Cutoff	GBA	GBA %	Control	Control %	p-value
**Transcranial sonography, cm²**	**Cutoff from literature**	≥ 0.2	31/157	19.7	12/46	26.1	0.412
	**10th percentile**	> 0.283	3/157	1.9	3/46	6.5	0.131
	**25th percentile**	> 0.2075	27/157	17.2	11/46	23.9	0.389
	**Outliers from GBA**	> 0.287	1/157	0.6	3/46	6.5	0.037
	**Above Z- score = 1.64**	> 0.25	11/157	7.0	6/46	13.0	0.194
	**Above Z-score = 1.96**	> 0.274	7/157	4.5	5/46	10.9	0.105
**Color discrimination test (TES)**	**Cutoff from literature**	Age 40-49 years > 100<br>Age 50-59 years > 130<br>Age 60-69 years > 170<br>Age 70-79 years > 195	2/154	1.3	0/44	0.0	1.000
	**10th percentile**	> 101.5	17/154	11.0	4/44	9.1	0.711
	**25th percentile**	> 65.5	37/154	24.0	11/44	25.0	0.894
	**Outliers**	> 125	5/154	3.2	1/44	2.3	0.868
	**Above Z- score = 1.64**	≥ 106	13/154	8.4	3/44	6.8	0.972
	**Above Z-score = 1.96**	≥ 125	6/154	3.9	1/44	2.3	0.959
**UPSIT- smell test**	**Cutoff from literature**	< 8	23/162	14.2	6/48	12.5	0.765
	**10th percentile**	< 7	13/162	8.0	2/48	4.2	0.362
	**25th percentile**	< 9	36/162	22.2	9/48	18.8	0.607
	**Outliers**	< 6	10/162	6.2	0/48	0.0	0.121
	**Above Z- score = 1.64**	< 8	23/162	14.2	6/48	12.5	0.765
	**Above Z-score = 1.96**	< 7	13/162	8.0	2/48	4.2	0.362
**UPSIT percentile**	**Cutoff from literature**	None	None	None	None	None	
	**10th percentile**	< 3.9	18/161	11.2	4/48	8.3	0.573
	**25th percentile**	< 14	39/161	24.2	11/48	22.9	0.852
	**Outliers**	None	None	None	None	None	
	**Above Z- score = 1.64**	None	None	None	None	None	
	**Above Z-score = 1.96**	None	None	None	None	None	
**Orthostatic hypotension**	**Cutoff from literature**	> 20 SBP or > 10 mmHg DBP	30/146	20.5	10/40	25.0	0.544

	**10th percentile**	> 13.9 or > 15.9	30/146	20.5	8/40	20.0	0.939
	**25th percentile**	> 8.75 or > 10.75	74/146	50.7	17/40	42.5	0.359
	**Outliers**	> 23 or > 19	6/146	4.1	1/40	2.5	0.996
	**Above Z- score = 1.64**	> 14 or > 16	24/146	16.4	6/40	15.0	0.827
	**Above Z-score = 1.96**	> 15 or > 18	16/146	11.0	4/40	10.0	0.909
**Bowel movement (daily)**	**Cutoff from literature**	≤ 0.5	10/158	6.3	5/49	10.2	0.361
	**10th percentile**	< 0.5	6/158	3.8	3/49	6.1	0.767
	**25th percentile**	< 1	21/158	13.3	0.2	16.3	0.593
	**Outliers**	< 1	21/158	13.3	0.2	16.3	0.593
	**Above Z- score = 1.64**	None	None	None	None	None	
	**Above Z-score = 1.96**	None	None	None	None	None	

(b) Cognitive and mental		**Cutoff**	**GBA**	**GBA %**	**Control**	**Control %**	**p-value**
**Beck depression inventory**	**Cutoff from literature**	≥ 14	12/154	7.8	3/47	6.38	0.996
	**10th percentile**	> 10.2	20/154	13.0	4/47	8.51	0.568
	**25th percentile**	> 8	26/154	16.9	9/47	19.15	0.720
	**Outliers**	> 14	12/154	7.8	2/47	4.26	0.613
	**Above Z- score = 1.64**	> 11	19/154	12.3	4/47	8.51	0.646
	**Above Z-score = 1.96**	> 14	15/154	9.7	2/47	4.26	0.377
**Frontal assessment battery**	**Cutoff from literature**	< 16	1/137	0.7	0/28	0.00	1.000
	**10th percentile**	< 17.9	25/137	18.2	2/28	7.14	0.243
	**25th percentile**	< 18	25/137	18.2	2/28	7.14	0.243
	**Outliers**	< 18	25/137	18.2	2/28	7.14	0.243
	**Above Z- score =1.64**	< 18	25/137	18.2	2/28	7.14	0.243
	**Above Z-score = 1.96**	< 18	25/137	18.2	2/28	7.14	0.243
**MoCA -Total score**	**Cutoff from literature**	≤ 25	37/157	23.6	9/45	20.00	0.615
	**10th percentile**	< 24	10/157	6.4	3/45	6.67	0.785
	**25th percentile**	< 26	37/157	23.6	9/45	20.00	0.615
	**Outliers**	< 22	1/157	0.6	0/45	0.00	1.000
	**Above Z- score = 1.64**	< 24	10/157	6.4	3/45	6.67	0.785
	**Above Z-score = 1.96**	< 24	10/157	6.4	3/45	6.67	0.785
**MoCA - Visuospatial/ executive**	**Cutoff from literature**	≤ 3	38/155	24.5	10/45	22.22	0.751
	**10th percentile**	< 3	10/155	6.5	2/45	4.44	0.887

	**25th percentile**	< 4	38/155	24.5	10/45	22.22	0.751
	**Outliers**	< 3	10/155	6.5	2/45	4.44	0.887
	**Above Z- score = 1.64**	< 3	10/155	6.5	2/45	4.44	0.887
	**Above Z-score = 1.96**	< 3	10/155	6.5	2/45	4.44	0.887
**Neurotrax - Memory**	**Cutoff from literature**	< 85	12/166	7.2	4/49	8.16	0.928
	**10th percentile**	< 85.6	13/166	7.8	4/49	8.16	0.822
	**25th percentile**	< 95.75	37/166	22.3	12/49	24.49	0.747
	**Outliers**	< 81.1	9/166	5.4	3/49	6.12	0.868
	**Above Z- score = 1.64**	< 84.4	10/166	6.0	4/49	8.16	0.838
	**Above Z-score = 1.96**	< 81.1	9/166	5.4	3/49	6.12	0.868
**Neurotrax - Executive function**	**Cutoff from literature**	< 85	6/166	3.6	2/49	4.08	0.781
	**10th percentile**	< 89	10/166	6.0	4/49	8.16	0.839
	**25th percentile**	< 98.35	32/166	19.3	12/49	24.49	0.427
	**Outliers**	< 78.7	2/166	1.2	1/49	2.04	0.799
	**Above Z- score = 1.64**	< 88.2	8/166	4.8	4/49	8.16	0.588
	**Above Z-score = 1.96**	< 84.9	5/166	3.0	2/49	4.08	0.930
**Neurotrax - Attention**	**Cutoff from literature**	< 85	5/166	3.0	2/49	4.08	0.930
	**10th percentile**	< 88.5	8/166	4.8	5/49	10.20	0.165
	**25th percentile**	< 97.1	25/166	15.1	12/49	24.49	0.124
	**Outliers**	< 86.4	6/166	3.6	3/49	6.12	0.716
	**Above Z- score = 1.64**	< 86.5	8/166	4.8	3/49	6.12	0.996
	**Above Z-score = 1.96**	< 83.9	3/166	1.8	2/49	4.08	0.697
**Neurotrax - Information processing**	**Cutoff from literature**	< 85	14/165	8.5	2/47	4.26	0.512
	**10th percentile**	< 87.38	20/165	12.1	4/47	8.51	0.668
	**25th percentile**	< 96.6	52/165	31.5	11/47	23.40	0.283
	**Outliers**	None	None	None	None	None	
	**Above Z- score = 1.64**	< 83.9	10/165	6.1	2/47	4.26	0.909
	**Above Z-score = 1.96**	< 80.1	3/165	1.8	1/47	2.13	0.641
**Neurotrax - Visual spatial**	**Cutoff from literature**	< 85	19/166	11.4	5/49	10.20	0.808
	**10th percentile**	< 84.8	19/166	11.4	4/49	8.16	0.696
	**25th percentile**	< 94.8	38/166	22.9	11/49	22.45	0.948
	**Outliers**	< 67	3/166	1.8	0/49	0.00	1.000
	**Above Z- score = 1.64**	< 83	17/166	10.2	3/49	6.12	0.554
	**Above Z-score = 1.96**	< 76.4	13/166	7.8	0/49	0.00	**0.043**
	**Cutoff from literature**	< 85	13/166	7.8	8/49	16.33	0.078

**Neurotrax - Verbal function**	**10th percentile**	< 61.8	6/166	3.6	4/49	8.16	0.346
	**25th percentile**	< 89.4	16/166	9.6	12/49	24.49	**0.007**
	**Outliers**	< 78.2	12/166	7.2	7/49	14.29	0.126
	**Above Z- score = 1.64**	< 62	6/166	3.6	5/49	10.20	0.067
	**Above Z-score = 1.96**	< 57	5/166	3.0	3/49	6.12	0.561
**Neurotrax - Motor skills**	**Cutoff from literature**	< 85	3/164	1.8	2/47	4.26	0.674
	**10th percentile**	< 95.54	18/164	11.0	4/47	8.51	0.828
	**25th percentile**	< 100.8	37/164	22.6	11/47	23.40	**0.032**
	**Outliers**	< 85.7	3/164	1.8	2/47	4.26	0.674
	**Above Z- score = 1.64**	< 92.1	10/164	6.1	3/47	6.38	0.785
	**Above Z-score = 1.96**	< 89.6	7/164	4.3	2/47	4.26	0.685
**Neurotrax - Global cognitive score**	**Cutoff from literature**	< 85	4/166	2.4	1/49	2.04	0.697
	**10th percentile**	< 88.6	4/166	2.4	4/49	8.16	0.150
	**25th percentile**	< 98.15	37/166	22.3	12/49	24.49	0.747
	**Outliers**	< 83.1	2/166	1.2	1/49	2.04	0.800
	**Above Z- score = 1.64**	< 89.4	4/166	2.4	5/49	10.20	0.047
	**Above Z-score = 1.96**	< 89.4	4/166	2.4	5/49	10.20	0.047

(c) Sleeping disorder		**Cutoff**	**GBA**	**GBA %**	**Control**	**Control %**	**p-value**
**REM sleep behavior disorder**	**Cutoff from literature**	≥ 5	18/156	11.5	11/48	22.9	**0.048**
	**10th percentile**	> 5.1	12/156	7.7	4/48	8.3	0.874
	**25th percentile**	> 4	18/156	11.5	11/48	22.9	**0.048**
	**Outliers**	> 7	5/156	3.2	0/48	0.0	0.593
	**Above Z- score = 1.64**	> 5	12/156	7.7	4/48	8.3	0.871
	**Above Z-score = 1.96**	> 6	7/156	4.5	1/48	2.1	0.745
**Epworth sleepiness scale**	**Cutoff from literature**	> 10	24/161	14.9	4/48	8.3	0.351
	**10th percentile**	> 10.1	24/161	14.9	4/48	8.3	0.351
	**25th percentile**	> 7.75	41/161	25.5	12/48	25.0	0.948
	**Outliers**	> 15	5/161	3.1	1/48	2.1	0.904
	**Above Z- score = 1.64**	> 11	18/161	11.2	3/48	6.3	0.469
	**Above Z-score = 1.96**	> 12	15/161	9.3	3/48	6.3	0.71

(d) Motor skills		**Cutoff**	**GBA**	**GBA %**	**Control**	**Control %**	**p-value**

**Perdue pegboard**	**Cutoff from literature**	< 11	2/159	1.3	0/46	0.0	1.000
	**10th percentile**	< 15.13	14/159	8.8	4/46	8.7	0.785
	**25th percentile**	< 17.25	35/159	22.0	11/46	23.9	0.786
	**Outliers**	None	None	None	None	None	
	**Above Z- score = 1.64**	< 14	7/159	4.4	3/46	6.5	0.842
	**Above Z-score = 1.96**	< 13.66	6/159	3.8	2/46	4.3	0.799
**UPDRS-III**	**Cutoff from literature**	> 10	7/143	4.9	1/40	2.5	0.828
	**10th percentile**	> 6	25/143	17.5	3/40	7.5	0.193
	**25th percentile**	> 3.75	55/143	38.5	10/40	25.0	0.116
	**Outliers**	> 11	3/143	2.1	1/40	2.5	0.647
	**Above Z- score = 1.64**	> 6	25/143	17.5	3/40	7.5	0.193
	**Above Z-score = 1.96**	> 7	21/143	14.7	3/40	7.5	0.355
