# Supplementary material for: Prodromal Parkinsonian Features in Carriers of Gaucher Disease Compared to Controls
Source: Life (Basel). 2025 Jun 13;15(6):952. doi: 10.3390/life15060952 (PMC12194337; doi:10.3390/life15060952)
Supplement: Supplementary file 1 [file life-15-00952-s001.zip › Supplement Table S2.pdf]

Table S2- PCA comparing subgroups in GBA1 carriers vs. controls

GBA1- carriers	Mean dif. (95% CI)	P-value PCA1	Higher score	Mean dif. (95% CI)	P-value PCA2	Higher score	Mean dif. (95% CI)	P-value PCA3	Higher score
N370S vs. non-N370S	0.451 (-0.211, 0.472)	0.466	NS	0.527 (-0.232, 0.451)	0.529	NS	0.342 (-0.506, 0.177)	0.292	NS
Patients GD vs. GBA1-carri	-0.264 (-0.619, 0.091)	0.144	NS	0.136 (-0.637, 0.109)	0.415	NS	0.124 (-0.232, 0.481)	0.507	NS
Age (< 55 vs. ≥ 55)	0.264 (-0.051, 0.579)	0.099	NS	0.925 (0.641, 1.208)	**< 0.001**	**Younger**	-0.29 (-0.05, 0.025)	0.071	NS
Family history of PD	0.413 (0.075, 0.751)	**0.017**	**No family history**	0.161 (-0.171, 0.492)	0.339	NS	-0.121 (-0.465, 0.233)	0.459	NS
Sex	0.539 (0.240, 0.838)	**0.001**	**Males**	-0.526 (-0.825, -0.266)	**< 0.001**	**Females**	0.305 (0.001, 0.609)	**0.049**	**Males**
Control									
Age (< 55 vs. ≥ 55)	0.079 (-0.503, 0.660)	0.787	NS	0.585 (0.029, 1.141)	**0.04**	**Younger**	0.657 (0.107, 1.206)	**0.02**	**Younger**
Family history of PD	0.28 (-0.357, 0.918)	0.381	NS	0.219 (-0.421, 0.858)	0.495	NS	0.033 (-0.610, 0.676)	0.918	NS
Sex	0.174 (-0.405, 0.754)	0.548	NS	0.339 (-0.912, 0.235)	0.241	NS	0.138 (-0.719, 0.442)	0.634	NS
